# The role of local business employees and community members in the HIV risk environment of female sex workers in an urban setting: associations between negative interactions and inconsistent condom use

**DOI:** 10.1186/s12889-021-12293-4

**Published:** 2021-12-11

**Authors:** Susan G. Sherman, Catherine Tomko, Bradley E. Silberzahn, Rebecca Hamilton White, Danielle Friedman Nestadt, Emily Clouse, Katherine Haney, Noya Galai

**Affiliations:** 1 Health, Behavior, and Society, Johns Hopkins Bloomberg School of Public Health, 624 N. Broadway St., Hampton House 180, Baltimore, MD 21205 USA; 2 Sociology, The University of Texas at Austin, 305 E 23rd St, A1700, RLP 3.306, Austin, TX 78712 USA; 3 Epidemiology, Johns Hopkins Bloomberg School of Public Health, 615 N. Wolfe St, Baltimore, MD 21205 USA

**Keywords:** Female sex work, Risk environment, Condom use, HIV

## Abstract

**Background:**

The role of business employees and community members in the HIV risk environment of female sex workers (FSW) is underexplored, despite sex work often located in commercial and residential urban areas. We explored the effect of negative interactions between business employees and community members on inconsistent condom use with clients of female sex workers.

**Methods:**

This study uses baseline data from the EMERALD study, a community empowerment intervention with FSW. We recruited a sample of 361 FSW in Baltimore, Maryland using targeted sampling techniques in ten zones characterized by high rates of sex work, located throughout the city. Participants were recruited between September 2017 and January 2019 and completed a survey, HIV rapid testing, and self-administered gonorrhea and chlamydia testing. The outcome, inconsistent condom use, was defined as not reporting “always” using condoms with paying clients. Poisson regressions with robust variance were used to model the effect of business employee and/or community member interactions on inconsistent condom use.

**Results:**

Over half (54%) the sample was between 18 and 40 years old, 44% Black or another race, and experienced a range of structural vulnerabilities such as housing instability and food insecurity. Forty-four percent of the sample reported inconsistent condom use with clients. FSW reported being reported to the police weekly or daily for selling drugs (14% by employees, 17% by community), for selling sex (19% by employees, 21% by community), and experiencing weekly or daily verbal or physical threats (18% by employees, 24% by community). In multivariable models, being reported to the police for selling sex weekly or daily by community members (vs. never, aRR = 1.42, 95% CI = 1.08, 1.86) and business owners (vs. never, aRR = 1.36, 95% CI = 1.05, 1.76) increased risk of inconsistent condom use, as did monthly verbal or physical threats by community members (vs. never, aRR = 1.43, 95% CI = 1.08, 1.91).

**Conclusions:**

Results show that both actors play important roles in FSWs’ HIV risk environment. Businesses and community members are important targets for holistic HIV prevention interventions among FSW in communities where they coexist in close proximity.

**Supplementary Information:**

The online version contains supplementary material available at 10.1186/s12889-021-12293-4.

## Background

A growing body of evidence has identified a complexity of factors that impact the HIV risk of female sex workers (FSWs) in their work environments [1–5]. Over the last decade, physical, social, economic, and policy environmental features have been articulated and examined which both constrain and enable risks and disease acquisition, including HIV and STIs [6–9]. Yet little is known about the role of business owners and community members in shaping HIV and STI risk among these women. Given their close proximity to street-based sex work venues, their potential influence merits examination.

Rhodes’ risk environment provides a useful framework as it is a theoretical approach that shifts the attribution of risk from that of the individual to the broader society, structures, and contexts in which they live and work [10, 11]. The framework suggests that behaviors and associated risks are positioned and influenced by the context in which they occur. These contexts can produce, heighten, or minimize individuals’ risks, bringing focus on the ways in which structures and individual agency interact to produce or minimize risk and harm [12]. Accordingly, health is tied to social conditions and structural factors rather than individual actions alone [11, 13]. This framework is rooted in a line of research that posits the dynamic interplay of individuals and their environments, which are characterized by complex systems that are dynamic and interactive [14–16]. Even in the context of harmful environments, individual agency or empowerment can alter the impact and nature of these settings or external structures.

Occupational environments for sex work are not equal, with vastly different risks profiles and sense of individual and collective agency associated with indoor compared to outdoor sex work settings. Indoor managed venues have been found to be associated with increased agency over negotiating sex work [9], lower violence exposure [9–11], and reduced HIV and STI risks [10, 11] compared to outdoor settings among sex workers in Canada. A diversity of environmental features of sex work have been implicated in drug and sexual risk for female sex workers (FSW), with police driving some of the most significant and egregious risks [4, 6, 17–19]. In the context of sex work criminalization as in the U.S., police are a key feature of FSWs’ HIV risk environment, with their impact intensified on those who work on the street [4, 19, 20]. Studies have documented significant associations between egregious police behaviors (e.g., sexual coercion, extortion) and HIV/STI infection [21–24]. Throughout Baltimore, street-based sex work predominantly occurs in mixed commercial and residential neighborhoods where FSW routinely encounter local residents and business employees in the course of selling sex. Further, community’s complaints and police responses can further “spatially marginalize” FSW, often moving them to sell sex in unfamiliar locations [25]. The physical isolation of the practice of street-based FSW has been associated with violence, unsafe sex [26, 27]; Yet little is known about the nature of FSWs’ engagement with these key actors within their occupational environments.

Additionally, police verbal harassment or sexual and physical assault have been associated with an increased risk of inconsistent condom use with clients and client violence [4, 22, 28–30]. Police attention to sex work in neighborhoods largely is in response from community complaints. Yet this initial step in the cascade of harms from police and clients, has been omitted from studies of contextual risk among this population. Recourse for police and client violence and other egregious behaviors is eclipsed by the illegal nature of sex work, functionally limiting women’s negotiating power that can often result in condom coercion [29–35]. The impact of these and other environmental features are amplified in the context of structural vulnerabilities including limited economic opportunities, housing instability, hunger, and violence as well as drug addiction [36–38].

In urban areas in the U.S., the street-based sex work scene is located in residential or commercial areas where businesses or community residents regularly interact with FSW [39, 40]. Residents of these areas cite moralized concerns about the reputation of the neighborhood or potential increase in violent crime as reasons for anti-sex work sentiments, though these sentiments are largely owing to stereotyping rather than experience [39, 41, 42]. This study aims to fill this gap in our understanding of the complexity of the risk environment’s impacts on FSWs’ health, we examine community members’ and police’s impact on sexual risk, namely inconsistent condom use.

## Methods

Data for this analysis are derived from the baseline EMERALD study, an evaluation of a HIV prevention, community-level intervention among street-based FSW in Baltimore, Maryland [43]. The intervention includes a full-service drop-in Center that provides medical, addiction, mental health, social service, and legal services as well as drop-in services (e.g., showers, lockers, laundry, food, a place to rest, therapeutic art groups, yoga). A detailed description of the study protocol and intervention have been published previously [43]. The EMERALD study is a prospective comparative intervention study with a baseline visit and three additional follow-up visits at 6-, 12-, and 18-months. Recruitment locations were identified through a targeted sampling process that we have updated in prior research with street-recruited FSW [44]. Briefly, relevant secondary data including 911 calls for prostitution and arrest data (e.g., solicitation, illicit drugs) were mapped and the occurrence of sex work was verified through window tours at different times of the day. This process resulted in a list of venues and associated times for recruitment at specific locations in 10 “zones” of sex work activity throughout Baltimore City. A mobile van was used to recruit FSW in these zones, six of which were in the intervention area where the SPARC drop-in center is location in West Baltimore, and four zones were in the control areas in other areas of the city. Bivariate analysis showed very few significant baseline differences between intervention and comparison areas in terms of demographics, sex work characteristics, and key study outcomes; the comparison area has a slightly higher percentage of Black participants than the intervention, but this association was marginal (*p* = 0.04*)*. Data from the Baltimore Neighborhood Indicators Alliance shows similar numbers of commercial properties, businesses, and households in areas comprising the intervention and comparison areas [45].

EMERALD eligibility criteria included: 1) aged 18 or older; 2) cis-gender woman; 3) sold or traded oral, vaginal, or anal sex “for money or things like food, drugs, or favors (past 3 months);” 4) picked up clients 3 or more times (past 3 months); and 5) willing to provide contact information for follow up visits. Eligible participants provided written informed consent, a 50-min Audio Computer-Assisted Self-Interview (ACASI) survey consisting of demographics, sex work history, drug use, and psychosocial measures. The survey was based on our previous research with the study population [21]. Participants also completed a rapid oral HIV test (OraQuick) and provided a self-administered vaginal swab which was sent to the Baltimore City Department of Health for gonorrhea and chlamydia testing. The study was approved by the Johns Hopkins Bloomberg School of Public Health Institutional Review Board.

EMERALD recruitment occurred in many of the same neighborhoods where we had previously conducted an observational study of FSW in which we employed similar recruitment methods [46]. As such, we had established connections with relevant stakeholders and established procedures for community engagement in advance of initiating data collection including: talking about the study in advance of recruitment with FSWs who sold sex on the street during the early stages of targeted sampling; contacting the leadership of and attending neighborhood community association meetings; and informing the local police management. We also discussed the study in its infancy with local FSW advocacy and harm reduction service organizations, all of whom we had existing relationships. Several study staff had lived sex work experiences that contributed to the overall relevance and sensitivity of study procedures and data collection instruments.

### Outcome

Participants were asked separately about frequency of recent (past week) condom use (“from the beginning to end of penetration”) during vaginal or anal sex with new and regular clients. Responses included: never, rarely, sometimes, most of the time, always, and did not have (vaginal/anal) sex. Inconsistent condom use (ICU) was defined as reporting any frequency other than “always” during vaginal or anal sex with new or regular clients.

### Independent variables

Demographics (e.g., age, ethnicity) and structural vulnerability context factors including homelessness, experiencing hunger (“because there was not enough food to eat”) at least weekly vs. less than weekly, financial dependence on someone, having financial dependent(s), and sources of income. We measured symptoms of post-traumatic stress disorder (PTSD) with the PTSD Patient Checklist (PCL-5) (scores > 33 indicated clinically significant PTSD symptoms) and depression with the Patient Health Questionnaire-9 (scores > 10 indicated presence of depressive symptoms) and recent (past 6 months) substance use include injecting any drug and using crack or powdered cocaine or heroin via any method [47, 48].

Sex work history variables included age started selling sex, frequency of sex exchange in past 6 months (daily vs. else), location(s) where women picked up new clients in the past 6 months, number of clients per week (split at the median, 1–5 vs. > 6), and if “someone takes a cut” of their sex work earnings. We also measured FSW responses to police practices in the past 6 months including rushing negotiations with clients due to police in the area, avoiding carrying condoms, or moving to an unfamiliar area to work to avoid police. Recent client-perpetrated physical or sexual violence was measured using an adapted version of the Revised Conflict Tactic Scale [49].

### Community member and business employees interactions

Participants reported the frequency, if ever, of types of interactions with business employees and community members, including: a) reporting them to the police for using drugs; b) asking them to leave an area in which they are selling sex; c) reporting them to police for selling sex; and d) verbally or physically threatening them. The eight response options ranged from never to every day and were collapsed into three categories: never, monthly, and weekly or daily. One binary yes/no question assessed whether women “rushed negotiations in the last 6 months because of (business employees/community members) in the area.” All questions were asked separately of business employees and community members.

### Statistical analysis

The sample size is 361 women. The analysis excluded 24 participants, 15 of whom did not answer questions related to community member and business employees questions and 9 who reported not engaging in vaginal and anal sex with new and regular clients. We used Pearson’s χ^2^ tests to evaluate differences in business employees or community members interactions, and other covariates by ICU. We used Poisson regressions with robust variance estimates for univariate regressions to determine unadjusted risk ratios of ICU by employee and community interactions and other covariates. Covariates at the *p* < 0.20 level were considered in subsequent multivariable models. We then used Poisson regressions with robust variance estimates for three separate multivariable models: Model A includes only business employee variables and covariates, Model B includes only community member variables and covariates, and Model C includes both business employee and community member variables and covariates. We used Akaike information criterion (AIC), Bayesian information criterion (BIC), and variance inflation factors to select the best fitting models and retained any covariates *p* < 0.15 in the final models. We fit models A, B, and C separately, though the same covariates emerged as significant in each. Significance was set at alpha = 0.05. All analyses were conducted in Stata/SE 15.1 (College Station, TX).

## Results

The cohort description with demographic, structural vulnerability, substance use, mental health, and sex work histories are reported in Table 1, stratified by reporting inconsistent condom use. Over two-thirds of the sample (65%) was between 18 and 39 years old and 57% was white (Table 1). A majority (67%) reported experiencing recent homelessness, 59% reported experiencing hunger at least weekly, and about half reporting clinically-significant PTSD (49%) and depressive (46%) symptoms. Most women (59%) sold sex for longer than 10 years, and over half (52%) sold sex daily. Gonorrhea or chlamydia prevalence at baseline was 15 and 21%, respectively, and HIV prevalence was 6%.

**Table 1:** Sample characteristics stratified by inconsistent condom use with clients among *n* = 361 female sex workers (FSW) in Baltimore, Maryland

	No Inconsistent Condom Use (*n* = 201)	Inconsistent Condom Use (*n* = 160)	Total (n = 361)	P
Demographics				
Age				0.37
*18–29*	45 (22.4)	39 (24.4)	84 (23.3)	
*30–39*	86 (42.8)	57 (35.6)	143 (39.6)	
*40–49*	52 (25.9)	42 (26.3)	94 (26.0)	
*50+*	18 (9.0)	22 (13.8)	40 (11.1)	
Education: 3 level				0.92
*<HS grad*	92 (45.8)	73 (45.6)	165 (45.7)	
*HS grad/GED*	52 (25.9)	39 (24.4)	91 (25.2)	
*Some col +*	57 (28.4)	48 (30.0)	105 (29.1)	
Race				0.45
*White*	116 (57.7)	86 (53.8)	202 (56.0)	
*Non-white*	85 (42.3)	74 (46.3)	159 (44.0)	
Arrested, past 6 months*	43 (21.7)	50 (31.4)	93 (26.1)	0.04
Economic Context				
Homeless, past 6 months	134 (66.7)	105 (65.6)	239 (66.2)	0.32
Experienced hunger…				0.32
*Less than weekly*	82 (40.8)	57 (35.6)	139 (38.5)	
*At least weekly*	119 (59.2)	103 (64.4)	222 (61.5)	
Depend on someone financially	146 (72.6)	127 (79.4)	273 (75.6)	0.15
Others depend on you financially	95 (47.3)	79 (49.4)	174 (48.2)	0.69
Sex work only source of income	53 (26.4)	41 (25.6)	94 (26.0)	0.87
Mental Health				
Clinically-significant PTSD symptoms*	96 (49.0)	93 (58.5)	189 (53.2)	0.07
Clinically-significant depressive symptoms*	92 (46.2)	74 (46.8)	166 (46.5)	0.91
Substance Use				
Used crack/cocaine, past 6 months	178 (88.6)	138 (86.3)	316 (87.5)	0.51
Used heroin, past 6 months	166 (82.6)	123 (76.9)	289 (80.1)	0.18
Injected drugs, past 6 months	119 (59.2)	92 (57.5)	211 (58.4)	0.74
Sex Work History & Characteristics				
Time in sex work				0.99
< *10 years*	83 (41.3)	66 (41.3)	149 (41.3)	
*> 10 years*	118 (58.7)	94 (58.8)	212 (58.7)	
Age started sex work*				0.41
*< 18*	38 (19.0)	36 (22.8)	74 (20.7)	
*18–30 years*	119 (59.5)	83 (52.5)	202 (56.4)	
*30+ years*	43 (21.5)	39 (24.7)	82 (22.9)	
Sold sex daily	104 (51.7)	86 (53.8)	190 (52.6)	0.7
Found new clients on the street*	157 (78.1)	141 (88.7)	298 (82.8)	0.01
Client per week				
*1 to 5*	101 (50.3)	54 (33.8)	155 (42.9)	0.002
*6+*	100 (49.8)	106 (66.3)	206 (57.1)	
Someone takes a cut of your earnings	26 (12.9)	25 (15.6)	51 (14.1)	0.47
Gonorrhea positive	30 (15.4)	23 (14.8)	53 (15.1)	0.89
Chlamydia positive	41 (20.8)	23 (14.9)	64 (18.2)	0.16
Violence				
Forced or pressured sex from clients, past 6 months*	62 (30.8)	74 (46.5)	136 (37.8)	0.002
Client physical violence, past 6 months	54 (26.9)	66 (41.3)	120 (33.2)	0.002
Client sexual violence, past 6 months*	40 (19.9)	53 (33.3)	93 (25.8)	0.004
Response to Police Practices				
Rushed negotiations, past 6 months	85 (42.7)	77 (48.4)	162 (45.3)	0.28
Avoided carrying condoms, past 6 months	20 (10.0)	35 (21.9)	55 (15.3)	0.002
Moved to an unfamiliar area to work, past 6 months	29 (14.7)	35 (21.9)	64 (17.9)	0.08

*< 3% data missing

Prevalence of ICU with clients was 44%. Women who used condoms inconsistently were significantly more likely to have recently been arrested, found new clients on the street, have six or more clients per week, and reported having experienced recent forced or pressured sex, physical violence, and sexual violence from clients. They were also more significantly likely to avoid carrying condoms due to police in the area.

Table 2 displays frequencies of business employee or community member interactions stratified by inconsistent condom use. Twenty-eight percent of women said they were asked to leave the area in which they sold sex on a daily or weekly basis, 18% were verbally or physically threatened by employees daily or weekly, and 31% rushed negotiations with clients because of business employees in the area daily or weekly. Seventeen percent of women said they were reported to the police for using drugs on a daily or weekly basis, 27% reported being asked to leave the area in which they sold sex daily or weekly, and 36% rushed negotiations with clients because of community members in the area on a daily or weekly basis. Women reporting ICU were more likely to be reported to the police by business employees daily or weekly for using drugs (*p* = 0.02) or for selling sex (*p* = 0.03) compared to women who reported consistent condom use. Women reporting ICU were more likely to be reported to the police by community members daily or weekly for selling sex (*p* = 0.04) and to be verbally or physically threatened by community members daily or weekly (p = 0.03) compared to women who reported consistent condom use.

**Table 2:** Frequencies of business employee or community member interactions stratified by inconsistent condom use among n=361 female sex workers (FSW) in Baltimore, Maryland

	No Inconsistent Condom Use (n = 201)	Inconsistent Condom Use (n = 160)	Total (n = 361)	P	No Inconsistent Condom Use (*n* = 201)	Inconsistent Condom Use (*n* = 160)	Total (n = 361)	P
	Business Employees			Community Members				
Frequency of ________ reporting you to the police for using drugs								
Never	156 (80.0)	106 (67.5)	262 (74.4)	0.02*	139 (70.2)	96 (60.8)	235 (66.0)	0.17*
Monthly	16 (8.2)	24 (15.3)	40 (11.4)		30 (15.2)	31 (19.6)	61 (17.1)	
Weekly/Daily	23 (11.8)	27 (17.2)	50 (14.2)		29 (14.5)	31 (19.6)	60 (16.9)	
Frequency of ___________ asking you to leave the area that you are selling sex								
Never	105 (53.3)	71 (44.7)	176 (49.4)	0.22*	103 (51.2)	65 (40.6)	168 (46.5)	0.13
Monthly	43 (21.8)	37 (23.3)	80 (22.5)		48 (23.9)	46 (28.8)	94 (26.0)	
Weekly/Daily	49 (24.9)	51 (32.1)	100 (28.1)		50 (24.9)	49 (30.6)	99 (27.4)	
Frequency of _____________ reporting you to the police for selling sex								
Never	139 (69.5)	90 (56.3)	229 (63.6)	0.03*	125 (62.2)	80 (50.0)	205 (56.8)	0.04
Monthly	34 (17.0)	30 (18.8)	64 (17.8)		42 (20.9)	37 (23.1)	79 (21.9)	
Weekly/Daily	27 (13.5)	40 (25.0)	67 (18.6)		34 (16.9)	43 (26.9)	77 (21.3)	
Frequency of ___________ verbally or physically threatening you								
Never	140 (69.7)	103 (64.4)	243 (67.3)	0.36	132 (65.7)	87 (54.4)	218 (60.7)	0.03
Monthly	30 (14.9)	23 (14.4)	53 (14.7)		23 (11.4)	33 (20.6)	56 (15.5)	
Weekly/Daily	31 (15.4)	34 (21.3)	65 (18.1)		46 (23.9)	40 (25.0)	86 (23.8)	
Rushed negotiations with clients because of _______ in the area								
Ever	56 (27.9)	57 (35.6)	113 (31.3)	0.19	64 (31.8)	66 (41.3)	130 (36.0)	0.17

*< 3% of data missing

### Multivariable model with business employees (model a)

Model A examined the relationship between interactions with business employees and ICU, controlling for key psychosocial and sex work characteristics (Table 3). Compared to women who never experience business employees reporting them to police for selling sex, being reported to the police weekly or daily was associated with greater risk of ICU (aRR = 1.36, 95% CI = 1.05, 1.76, *p* = 0.02). In the presence of other variables, there were significant elevated risk of ICU in association with: finding new clients on the street (adjusted risk ratio (aRR) = 1.58, 95% confidence interval (CI) = 1.06, 2.35, *p* = 0.03), experiencing forced or pressured sex from clients (aRR = 1.30, 95% CI = 1.04, 1.63, *p* = 0.02), having six or more clients per week (compared to 1–5, aRR = 1.37, 95% CI = 1.07, 1.77, p = 0.02), and avoided carrying condoms because of police (aRR = 1.33, 95% CI = 1.02, 1.72, p = 0.03).

**Table 3:** Bivariate and multivariable Poisson models with robust variance modeling inconsistent condom use among *n* = 361 female sex workers (FSW) in Baltimore, Maryland

	Unadjusted Models		Model A		Model B		Model C	
	RR (95% CI)	p	aRR	p	aRR (95% CI)	p	aRR (95% CI)	p
***Business Employee Variables***								
Frequency of reporting you to the police for using drugs (ref = never)								
*Monthly*	1.48 (1.11, 1.99)	0.01	–	–			–	–
*Weekly/Daily*	1.35 (0.99, 1.79)	0.06	–	–			–	–
Frequency of reporting you to the police for selling sex (ref = never)								
*Monthly*	1.19 (0.88, 1.62)	0.26	1.06 (0.78, 1.43)	0.71			1.04 (0.75, 1.42)	0.83
*Weekly/Daily*	1.52 (1.18, 1.96)	0.001	**1.36 (1.05, 1.76)**	**0.02**			1.33 (0.99, 1.79)	0.06
Ever rushed negotiations with clients because of business owners (ref = never)	1.22 (0.97, 1.55)	0.09	–	–			–	–
***Community Member Variables***								
Frequency of reporting you to the police for selling sex (ref = never)					–	–	–	–
*Monthly*	1.20 (0.90, 1.61)	0.22			1.01 (0.75, 1.36)	0.96	0.99 (0.72, 1.35)	0.93
*Weekly/Daily*	1.45 (1.12, 1.88)	0.01			**1.42 (1.08, 1.86)**	**0.01**	1.26 (0.93, 1.70)	0.14
Frequency of verbally or physically threatening you (ref = never)								
*Monthly*	1.48 (1.13, 1.95)	0.01			**1.43 (1.08, 1.91)**	**0.01**	**1.44 (1.08, 1.90)**	**0.01**
*Weekly/Daily*	1.17 (0.89, 1.55)	0.27			0.88 (0.66, 1.18)	0.40	0.84 (0.62, 1.14)	0.27
Ever rushed negotiations with clients because of community members (ref = never)	1.25 (0.99, 1.58)	0.06			–	–	–	–
***Contextual Characteristics***								
Found new clients on the street (ref = no)		0.02		0.03		0.05		0.02
*Yes*	1.63 (1.08, 2.45)		1.58 (1.06, 2.35)		1.58 (1.07, 2.34)		1.58 (1.07, 2.34)	
Client forced or pressured sex (ref = no)		0.002		0.02		0.05		0.05
*Yes*	1.44 (1.14, 1.80)		1.30 (1.04, 1.63)		1.25 (1.00, 1.58)		1.26 (1.00, 1.58)	
Client per week (ref = 1–5)				0.01		0.01		0.01
*6+*			1.37 (1.07, 1.77)		1.39 (1.08, 1.78)		1.38 (1.08, 1.77)	
Avoided carrying condoms because of police (ref = no)		< 0.001		0.03		0.01		0.01
*Yes*	1.55 (1.22, 1.98)		1.33 (1.02, 1.72)		1.41 (1.10, 1.81)		1.37 (1.06, 1.76)	

### Multivariable model with community members (model B)

Model B examined the relationship between interactions with community members and ICU, controlling for key psychosocial and sex work characteristics. Compared to women who never experienced verbal or physical threats from community members, experiencing threats monthly was associated with greater risk of ICU (aRR = 1.43, 95% CI = 1.08, 1.91, *p* = 0.01), though experiencing threats weekly or daily was not significantly associated with ICU. Compared to women who never experience community members reporting them to police for selling sex, being reported to the police weekly or daily was associated with greater risk of ICU (aRR = 1.42, 95% CI = 1.05, 1.76, p = 0.01). Additionally, there was significant elevated risk of ICU in association with: finding new clients on the street (aRR = 1.58, 95% CI = 1.07, 2.34, *p* = 0.05), experiencing forced or pressured sex from clients (aRR = 1.25, 95% CI = 1.00, 1.58, p = 0.05), having six or more clients per week (compared to 1–5, aRR = 1.39, 95% CI = 1.08, 1.78, *p* = 0.01), and avoided carrying condoms because of police (aRR = 1.41, 95% CI = 1.10, 1.81, p = 0.01).

### Multivariable model with business employees and community members (model C)

Model C examined the relationship between interactions with business employees and community members, controlling for key psychosocial and sex work characteristics, which were similarly significant in this model as models A and B. Further, when including both business employee and community member interaction in the model, ICU was only associated with experiencing verbal or physical threats from community members on a monthly basis compared to never experiencing threats (aRR = 1.44, 95% CI = 1.08, 1.90, *p* = 0.01).

## Discussion

This study examines the negative impact of businesses and community members on sexual risk among a sample of FSWs recruited on the street. This is one of the first studies to identify these groups as social features of FSWs’ occupational risk environment. We found a significant association between negative interactions with both business and community members on inconsistent condom use with clients. This association indicates the degree to which these social aspects of women’s occupational risk environment can undermine their agency in self protection. Findings indicate the frequency and impact of these exchanges on women’s HIV and STI risk beyond salient structural, mental health, and sex work-related factors.

The frequency of interactions with businesses and community members was high. In separate models, being reported to the police frequently by both business employees and community members was significantly associated with inconsistent client use in the presence of key demographic and sex work characteristics. Frequent threats of verbal or physical abuse by community members were associated with inconsistent condom use with clients. Lastly, when business and community interactions were in the same model controlling for key relevant demographic and sex work characteristics, monthly verbal or physical threats from community members maintained significance. Overall, women reported more frequent interactions with community members compared to business owners, likely owing to their being in the neighborhood more often than business employees at night when women are working. Although not directly related to unprotected sex, these types of engagements with community members and businesses likely creates hostile work environments for FSW driving women to rush negotiations so that they have less possibility of police interaction or arrest if they were called.

FSW possess little recourse for physical or verbal threats or calls to the police given the criminalization of sex work. Engaging with the police, whether threats (e.g., verbal harassment) or actions (e.g., arrest, sexual violence) is a number of harmful outcomes [17, 30, 50]. In the current study, the impact of police was reflected in the significant association between client inconsistent condom use with both avoiding carrying condoms and moving to unfamiliar areas in all three adjusted models, both attributed to the police. An earlier study of ours regarding FSW in Baltimore found associations between abusive policing practices and client-perpetrated violence, which, in turn, has been found to increase risk for inconsistent condom use [4]. Sex work decriminalization effectively increase women’s agency and afford many protections to women including their ability to organize and negotiate around where they conduct their business.

The study sheds light on the role of these social aspects of FSW’s HIV risk environment, complimenting existing literature that examines the nature of the built environment and impact on FSWs’ agency as well as sexual and drug related risk profiles [9, 18]. Similar to street-based FSW in Baltimore and elsewhere in the US, reported rates of illicit drug use are high [17, 46]. Overlapping drug and sex markets likely impact communities synergistically, fueling animosity toward drug-using FSW and exacerbating their interactions with them [18]. Further, community members’ and businesses’ frustrations over the impact of the sex and drug economies on their neighborhoods could result in disproportionate responses on FSW compared to male who use drugs.

The current sample was characterized by several structural vulnerabilities, including low prevalence of education and high prevalence of homelessness, hunger, and arrest. Structural vulnerability refers to the position of an individual within social structures and the impact of those structures such as laws (e.g., illegality of prostitution or drug use), policing, and access to resources such as housing and employment [13]. The impact of these vulnerabilities in alone and tandem can render women less able to weather the impact of frequent negative interactions with businesses and community members. The occurrence of sex work in a given neighborhood can be perceived by residents and businesses as a driver for devaluating property values, placing disproportionate blame on these women for external economic, urban planning, and broader drug market trends [41, 51]. This misplaced anger may exacerbate FSW stigma, resulting in further marginalization and isolation of women in moving them to work in more concentrated, hidden, and dangerous spaces, thereby increasing their exposure to violence and risk [7, 18, 52].

A recent study examining business and community members opinions toward a proposed overdose prevention site (OPS) in their neighborhood is instructive. The study found high acceptability even in light of frustrations with public drug use in their neighborhood [53]. A similar study among businesses and community members could examine their attitudes towards sex workers, sex work in their neighborhood, and a safe sex work space parallel to an OPS. Results could inform sex worker stigma reduction interventions and eventually decriminalization educational and advocacy efforts, both of which would foster a less hostile occupational environment, which increase women’s ability to enact agency and reduce their experiences of a range of health risks [54].

The current study is characterized by several limitations. It is important to remember that these results are not reflective of all FSW. Our sample is recruited from the street and primarily, but not exclusively, find clients on the street; they are also characterized by structural vulnerabilities. Further, sex work is illegal in the United States and findings with respect to threats of police involvement are not likely generalizable to decriminalized sex work environments. Second, we collected data using ACASI software on tablet computers, which can provide more privacy than interviewer-administered surveys but limits the ability to probe unclear responses. Third, we did not define community members or business owners for participants before asking about those exposures, so there may be some variability in how individual participants defined these terms for themselves. Finally, data is self-reported and may be subject to desirability bias especially the outcome of client condom use.

## Conclusions

In summary, the results from our study highlight the importance of a features of the HIV risk environment in its direct and indirect negative impact on street-based FSW that have not previously been examined. Businesses and community members are salient environmental features of FSWs’ workspace, that should be included in holistic HIV prevention and occupational safety interventions for FSWs. The prevalence of negative interactions with these populations including being physically or verbally threatened or being reported to the police creates both a hostile work and impacts women’s sexual risk profile. The impact of sex work criminalization and stigma is far reaching, and in this case, tacitly giving license for maltreatment of sex workers by others who are perceived or actually impacted by their occupational location. Police more likely to protect business and residential interests than that of FSW in any of these encounters, with the tool of arrest not only failing to reduce sex work but also generating a number of harms. Future research is necessitated to understand the complexity of interactions between FSW and these actors. Further, research with businesses and community would provide insights to understand their perspectives and point to levers of change to both engage them in HIV prevention and occupational safety interventions.

## Supplementary Information

**Additional file 1.**

## Data Availability

The datasets analyzed during the current study are not publicly available due participant confidentiality but are available from the corresponding author on reasonable request.

## Abbreviations

FSW: female sex worker
HIV: human immunodeficiency virus
STI: sexually transmitted infection
ACASI: audio computer assisted self-interview
AIC: Akaike information criterion
BIC: Bayesian information criterion
PTSD: post-traumatic stress disorder
ICU: inconsistent condom use
RR: risk ratio
CI: confidence interval
OPS: overdose prevention site
