# Supplementary material for: The role of local business employees and community members in the HIV risk environment of female sex workers in an urban setting: associations between negative interactions and inconsistent condom use
Source: BMC Public Health. 2021 Dec 11;21:2265. doi: 10.1186/s12889-021-12293-4 (PMC8666055; doi:10.1186/s12889-021-12293-4)
Supplement: Supplementary file 1 — Additional file 1. [file 12889_2021_12293_MOESM1_ESM.docx]

June 10, 2021

We appreciate the reviewers’ thoughtfulness which has strengthened the manuscript. Below please find our responses that are indented below the reviewers’ specific comments. In our response, we reference the location of changed text and when appropriate, we include these changes to the manuscript displayed in italics. Reviewer 1 provided an initial paragraph, somewhat of a summary of ensuing comments, which were then followed by specific points. It seemed to us that the initial paragraph was a summary rather than being stand alone, so to be safe we responded to it in addition to the ensuing concerns, noting any overlap.

**Reviewer 1: Summary**

Major revisions and comments

Clarification on the study design and methods is needed to strengthen the results presented.

- We have enhanced our description of the study design by specifying that the longitudinal design was inclusive of the community empowerment intervention and I also added the number and spacing of follow-ups. The description (pg. 5) now reads, “*The EMERALD study is a prospective comparative intervention study with a baseline and three additional follow-up visits at 6-, 12-, and 18-months*.” This aligns with our description of EMERALD in other articles.
- Further, we have expanded our description of targeted sampling directly following the text describing the study design on pg. 5 which now reads,

*Recruitment locations were identified through a targeted sampling process that we have updated in prior research with street-recruited FSW.(45) Briefly, relevant secondary data including 911 calls for prostitution and arrest data (e.g., solicitation, illicit drugs) were mapped and the occurrence of sex work was verified through window tours at different times of the day. This process resulted in a list of venues and associated times for recruitment at specific locations in 10 “zones” of sex work activity throughout Baltimore City. A mobile van was used to recruit FSW in these zones, six of which were in the intervention area in close proximity to the drop-in center’s West Baltimore location, and four zones were in the control areas in other areas of the city.*

- We have also added a description of the comparability of the intervention and control area to the methods (pg. 5) below.

*Bivariate analysis showed very few significant baseline differences between intervention and comparison areas in terms of demographics, sex work characteristics, and key study outcomes; the comparison area has a slightly higher percentage of Black participants than the intervention, but this association was marginal (p=0.04). Data from the Baltimore Neighborhood Indicators Alliance shows similar numbers of commercial properties, businesses, and households in areas comprising the intervention and comparison areas (46).*

The authors ground their study design in the Risk Environment framework. There are updates to this framework that may be relevant, including iterations of this framework that account not only for structural vulnerability and marginalization, but also sex workers' agency. It would be nice for the authors to consider referencing this given the tremendous amount of agency that has also been demonstrated amongst sex workers in various settings (e.g., via the community empowerment literature).

- We appreciate the reviewer’s recommendation to enhance the risk environment framework in the Introduction to include sex workers’ agency, when appropriate, reflecting a more updated literature and additionally, a strengths-based representation. The reviewer’s comment informed our including a more expansive description of the interplay of environment and agency in both promoting or inhibiting risk, with several changes made in the first three paragraphs of the background section (pg. 3). While separate but related, their suggestion also prompted us to include a brief description of the differential risk and sense of agency associated with outdoor compared to indoor sex work settings on the bottom of pg. 3, providing a more nuanced understanding of the role of the environment in building the case for the analysis. The following text was added:

*All occupational environments for sex work are not equal, with vastly different risks associated with indoor compared to outdoor sex work settings. Indoor managed venues have been found to be associated with increased agency over negotiating sex work (9), lower violence exposure (9–11), and reduced HIV and STI risks (10,11) compared to outdoor settings among sex workers in Canada.*

- We also added nuance to the Discussion with more focus on agency in discussing the study findings. We added the following sentence to first paragraph in this section in discussing the study’s main finding (pg. 11), *“This association indicates the degree to which these social aspects of women’s occupational risk environment can undermine their agency in self protection.”* Further, we added the impact of decriminalization on increasing women’s agency at the bottom of pg. 12.
- We appreciate the reviewer’s recommendation for referencing discussions of agency from such literature as community empowerment. This literature is directly related to our community empowerment intervention evaluated in EMERALD. But for the current analysis, we felt that our discussion of agency in the aforementioned manner was more appropriate. Further, we now propose (described below) an intervention resulting from the study’s findings that focuses on community members and business owners, which in turn will impact women’s ability to enact agency. We describe this on pg. 15.

*Results could inform sex worker stigma reduction interventions and eventually decriminalization educational and advocacy efforts, both of which would foster a less hostile occupational environment, which increase women’s ability to enact agency and reduce their experiences of a range of health risks.*

- We did not intend to minimize women’s agency by our prior lack of focus and hope that our more explicit inclusion of agency adds nuance to our discussion of how negative police and community interactions function to inhibit women’s agency and impact health outcomes.

This study is positioned within the broader EMERALD which the authors note is described elsewhere. If feasible in terms of text space, a brief comment on how community engagement was operationalized and how recruitment was carried out would be welcome.

- We noted that the current analysis is from the EMERALD study and have now expanded our description of our recruitment methodology (targeted sampling), discussed above. We are unclear about the request to operationalize “community engagement” – in terms of how we engaged with the sex worker and/or neighborhood associations in the areas in which we conducted the study? Or the nature of community engagement with our drop-ion Center? We are happy to add any description once this is clarified.

The concept of gentrification is introduced early in the paper but is not a thread that carries through beyond the Background. The concept of structural vulnerability is discussed in the methods and would benefit from being in the Background section for consistency and unity of themes.

- We agree and have dropped the reference to gentrification as it is not relevant to the included neighborhoods and current analysis. We added a reference to structural vulnerabilities in the Introduction at the bottom of pg. 4 as follows. While this is mentioned without being defined or more fully discussed, we feel that naming it as an amplifier of the impact of deleterious environments is helpful in understanding why we use this language in the methods.

*The impact of these and other environmental features are amplified in the context of structural vulnerabilities including limited economic opportunities, housing instability, hunger, and violence as well as drug addiction. (36-38)*

Additional details on the study design and methods are needed to fully understand the context of this research including greater detail in defining the exposures of interest (e.g., how did the authors define what a business is? Who is a community member? Neighbourhood descriptions and demographics would be necessary to contextualize the findings). Better description between "intervention" and "comparison" areas is also needed.

- We did not give prescriptive definitions of business owners or community members and have added the following as a limitation in the Discussion section on pg. 14. Although this can be seen a limitation, we trusted women’s understanding of these actors particularly in relation to how they have been impacted by them.

*Third, we did not define community members or business owners for participants before asking about those exposures, so there may be some variability in how individual participants defined these terms for themselves.*

- We have added the following additional contextual information about sex work in Baltimore to the Introduction on pg. 4,

*Throughout Baltimore, street-based sex work predominantly occurs in mixed commercial and residential neighborhoods where FSW routinely encounter local residents and business employees in the course of selling sex.*

- As noted above, we added text (pg. 6) describing the comparability of the intervention and control areas as reflected in baseline data comparing women from the intervention and comparison areas and data from the Baltimore Neighborhood Indicators Alliance.

Clarification is requested regarding the measures and data presented; the focus of the paper and objectives don't seem to match the final models presented. For example, data on confounders was presented when it seems likely the authors are mainly querying businesses and community members exposure only. As an example, why was data presented regarding PTSD scores? This clarification will ensure the study objectives and the final methods are clearly aligned.

- We have edited Table 3 to remove contextual characteristic variables that were not included in the multivariable models and for which we only presented unadjusted results. This includes recent arrest, PTSD score, and changing location due to police presence.

Discussion of results and findings would be strengthened with more information of the interactions between the actors and female sex workers. For example, contextualize the legalities that shape how sex work is policed; are there specific municipal bylaws that are being used?

- We did not collect additional information about interactions between sex workers and community members or business members outside of the four that we reported in the current study. We specified that sex work is criminalized in the U.S. (pg. 4). We did not reference specific municipal by-laws as there are none that shape police enforcing the crime of selling sex by arrest that are distinct in Baltimore compared to anywhere else in the US (outside of Nevada where sex work is legal).

The authors ground their research in a Risk Environment framework, which is a strength; yet some points in the Discussion section to not support this. For example, better description of how the risk environment undermines sex workers' agency.

- We re-grounded the study focus on the risk environment as well as how women’s agency can be undermined by the environment by adding text in several places throughout the Discussion section including the following sentence in the first paragraph (pg. 11) in the Discussion section as well as several additional references to the relationship between environment and agency on pg. 12.

*This association indicates the degree to which these social aspects of women’s occupational risk environment can undermine their agency in self protection.*

Finally, the discussion regarding the overdose prevention site seems out of place and may require a bit more explanation to clarify how it fits with the broader arguments being made. More detailed description of the neighbourhoods of focus in this study would be helpful to contextualize the findings and help clarify how they may be relevant to other contexts. This in turn would strengthen the recommendation of targeting interventions to businesses/community members, as this may depend heavily on the local context and role these actors play within the local sex work landscape.

- The reference to OPS has been revised to enhance its relevance to the current study’s findings. The revision is located on pg. 14 and is as follows,

*A recent study examining business and community members opinions toward a proposed overdose prevention site (OPS) in their neighborhood is instructive. The study found high acceptability even in light of frustrations with public drug use in their neighborhood. (53) A similar study among businesses and community members could examine their attitudes towards sex workers, sex work in their neighborhood, and a safe sex work space parallel to an OPS. Results could inform sex worker stigma reduction interventions and eventually decriminalization educational and advocacy efforts.*

- The relevance of overdose prevention sites, also called safe consumption sites, is hopefully apparent in that both illicit drugs and illegal sex work are met with great community opposition, while some spaces, whether it be an OPS or decriminalizing sex work in a city, could be beneficial for and advocated by the very community members who are often in opposition.

Minor comments:

Some grammatical errors and inconsistency in language are present. For example:

*       Under "Conclusions" line 56-58 "being reported to police" is written twice in the same sentence

*       In the Abstract adjusted Odds Ratios are mentioned. Do the authors mean to write Risk Ratio?

- We apologize and have corrected these and other typos in the manuscript.

**Reviewer 2**

REQUESTED REVISIONS:

The title of the manuscript should be varied to reflect the main purpose of the study. In one way it seems the study is interested in the role of Local Business Employees and Community Members in the HIV Risk behaviours of Female Sex Workers (FSW), and in another angle, it looks like the authors are interested in inconsistent condom use pattern about FSW. Since the outcome variable in the main study is "inconsistent condom use", I will suggest that the authors frame the title as " Inconsistent condom use among Female Sex Workers in an Urban Setting: Does Local Business Employees and Community Members play a role? OR "The role of Local Business Employees and Community Members on Female Sex Workers' Inconsistent condom use."

- We agree that the previous title was confusing and have simplified it by shortening the original title to read as follows: “The Role of Local Businesses and Community Members in the HIV Risk Environment of Female Sex Workers in an Urban Setting.”

The last sentence in the background of the abstract should read "We explored the effect of negative interactions between business employees and community members on inconsistent condom use of female sex workers."

- We revised the sentence to reflect the proposed changes.

The statement " Compared to women who never experience community members reporting them to police for selling sex, being reported to the police weekly or daily was associated with greater odds of ICU (aRR=1.42, 95% CI=1.05, 1.76), which can be re-framed as " Female sex workers (FSW) who are reported by community members daily or weekly to the police for selling sex  had greater odds of ICU (aRR=1.42, 95% CI=1.05, 1.76, p =0.01)

- We appreciate the less convoluted sentence proposed by the reviewer, which could be applied in several instances. But we propose keeping this and other similar sentences for the purposes of thoroughness in providing the reference category when reporting results of the multivariate models.

To help the reader determine the magnitude of these risk ratios, the p values should be reported.

- We have added p-values for all multivariable models.

We appreciate your reconsidering the manuscript and feel that the revised version benefited from these reviews.

Sincerely,

Susan G. Sherman, PhD, MPH

Bloomberg Professor in American Health
